# The Neotropical endemic liverwort subfamily Micropterygioideae had circum‐Antarctic links to the rest of the Lepidoziaceae during the early Cretaceous

**DOI:** 10.1002/ece3.11066

**Published:** 2024-03-03

**Authors:** Antonio L. Rayos, Matthew A. M. Renner, Simon Y. W. Ho

**Affiliations:** ^1^ School of Life and Environmental Sciences University of Sydney Sydney New South Wales Australia; ^2^ Institute of Biological Sciences University of the Philippines Los Baños Los Baños Laguna Philippines; ^3^ National Herbarium of New South Wales Royal Botanic Gardens Sydney Sydney New South Wales Australia

**Keywords:** biogeography, Lepidoziaceae, liverworts, Micropterygioideae, *Micropterygium*, molecular dating, vicariance

## Abstract

Lepidoziaceae are the third‐largest family of liverworts, with about 860 species distributed on all continents. The evolutionary history of this family has not been satisfactorily resolved, with taxa such as Micropterygioideae yet to be included in phylogenetic analyses. We inferred a dated phylogeny of Lepidoziaceae using a data set consisting of 13 genetic markers, sampled from 147 species. Based on our phylogenetic estimate, we used statistical dispersal‐vicariance analysis to reconstruct the biogeographic history of the family. We inferred a crown age of 197 Ma (95% credible interval 157–240 Ma) for the family in the Australian region, with most major lineages also originating in the same region. Micropterygioideae are placed as the sister group to Lembidioideae, with these two lineages diverging from each other about 132 Ma in the South American–Australian region. With South America and Australia being connected through Antarctica at the time, our results suggest a circum‐Antarctic link between Micropterygioideae and the rest of the family. Crown Micropterygioideae were inferred to have arisen 45 Ma in South America before the continent separated from Antarctica. Extinction from southern temperate regions might explain the present‐day restriction of Micropterygioideae to the Neotropics. Our study reveals the influence of past geological events, such as continental drift, on the evolution and distribution of a widespread and diverse family of liverworts.

## Graphical abstract

We inferred the position of Micropterygioideae in the molecular phylogeny of the liverwort family Lepidoziaceae for the first time and found circum‐Antarctic links of the subfamily to the rest of the family. Additionally, we estimated the divergence times and inferred the ancestral ranges of the major clades of the family. The crown origin of the family before the fragmentation of Gondwana at least partially accounts for its cosmopolitan distribution.

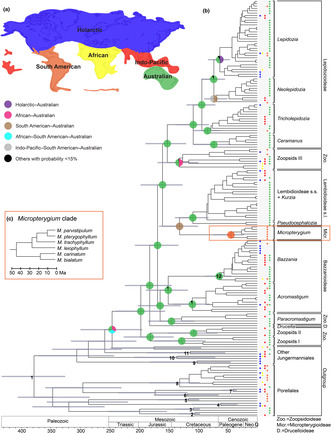

## 1 INTRODUCTION

Liverworts (Marchantiophyta), one of the three major groups of bryophytes, emerged soon after the colonisation of terrestrial habitats by plants during the Ordovician, but the lineage underwent a marked diversification during the early Paleogene (Simpson, 2010; Vanderpoorten & Goffinet, 2009). Among the most widely distributed families of liverworts are Lepidoziaceae, which are the third‐largest liverwort family and comprise about 860 species in 29–31 genera and seven subfamilies (Cooper, 2013; Cooper et al., 2011; Crandall‐Stotler et al., 2009). As with other diverse liverwort families, many genera of Lepidoziaceae are understood to have arisen in the early Cenozoic (Cooper et al., 2012b). Representatives of Lepidoziaceae occur on all continents, including Antarctica, and inhabit a wide variety of bioclimatic zones, habitat types and substrates, including soil, peaty ground, decaying wood and tree trunks.

While exhibiting an almost unparalleled diversity of form in the gametophyte generation, species of Lepidoziaceae are unified by a set of unique morpho‐anatomical characteristics of the sporophyte, including very small spores, elaters with blunt ends and two‐phase ontogeny of the capsule epidermis (Schuster, 1969, 2000). All members of the family also share isophyllous gynoecial branches (Schuster, 2000). Although the monophyly of the family sensu Schuster (2000) has been established with confidence, molecular phylogenetic studies have not completely resolved the evolutionary relationships among extant species.

The first molecular phylogenetic analysis of Lepidoziaceae used three organellar markers and revealed polyphyly in the subfamilies Zoopsidoideae, Lepidozioideae and Lembidioideae (Heslewood & Brown, 2007). Subsequently, analysis of a larger data set comprising 10 loci from 93 species confirmed the polyphyly of Lepidozioideae and Zoopsidoideae and recovered Lembidioideae as paraphyletic (Cooper et al., 2011). The polyphyly of Lepidozioideae motivated the transfer of *Kurzia* to Lembidioideae (Cooper, 2013). However, sequence data from 10 molecular markers did not allow confident estimation of the basal relationships among subfamilies of Lepidoziaceae, and these remain unresolved. In addition, several distinct taxa, including the subfamily Micropterygioideae, were not included in the molecular phylogenetic studies of Cooper et al. (2011, 2012a, 2012b), and their relationships remain opaque.

**FIGURE 1:**
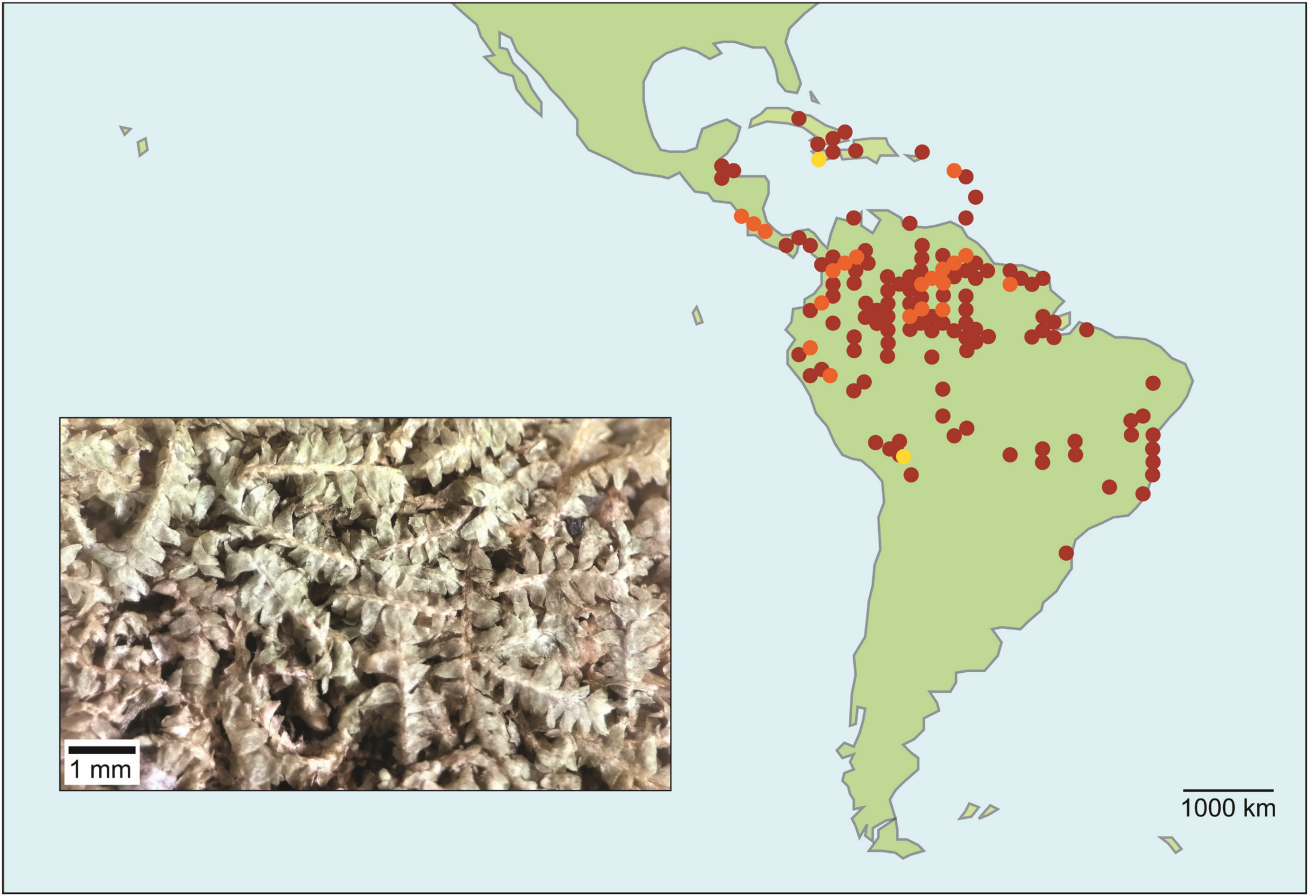
Geographic distribution of Micropterygioideae, with a photomicrograph of a representative species (*Micropterygium leiophyllum*). The red dots represent GBIF records of *Micropterygium* [GBIF.org (20 January 2024) GBIF Occurrence Download https://doi.org/10.15468/dl.gdsvum]; yellow dots, *Mytilopsis* [GBIF.org (20 January 2024) GBIF Occurrence Download https://doi.org/10.15468/dl.6eezj7]; and orange dots, both genera.

The Micropterygioideae are confined to the Neotropics, where species mostly occur in the lowlands, although some species reach the Andes (up to 3140 m above sea level). Most species are limited to the Amazonian drainage, but some extend to the Caribbean Islands. The subfamily contains two genera, *Micropterygium* and *Mytilopsis*, which differ from members of the other subfamilies in their conduplicate leaves that have an abaxial wing or ridge (Schuster, 1969, 2000). In some of their features, the subfamily has morphological similarities to some members of Lembidioideae. *Micropterygium* includes about 20 species (with a representative species, *M. leiophyllum*, shown in Figure 1) and differs from the monotypic *Mytilopsis* in having underleaves and lateral‐intercalary rather than ventral‐intercalary vegetative branches. Micropterygioideae might share a close relationship with Lembidioideae and, through their common ancestor, have connections to the “old cool‐Gondwana flora” (Schuster, 1992). Without knowing the phylogenetic relationship of Micropterygioideae to the rest of Lepidoziaceae, however, it is impossible to test hypotheses regarding its origins.

Lepidoziaceae show generic richness and high endemism concentrated in the circum‐Antarctic region, a pattern that has been explained by an origin, or at least a diversification, centred on Gondwana (Schuster, 2000). The confinement of Micropterygioideae to the Neotropics is unusual and could be explained by extinction of the lineage in the circum‐Antarctic region. Micro‐ and macrofossil evidence suggests that many vascular plant lineages currently confined to single southern temperate land masses were previously more widely distributed in southern temperate regions. For example, fossil Winteraceae are known in South Africa (Coetzee & Praglowski, 1988); *Casuarina* and *Eucalyptus*, in New Zealand (Mildenhall, 1980) and southern South America (Gandolfo et al., 2011); and *Dacrydium*, in Australia (Keppel et al., 2011). In the New Zealand context, extinctions might have been caused by a transition to colder and wetter climatic conditions and Pleistocene glacial‐interglacial cycling (Lee et al., 2001). The latter is likely to have had an impact on southern South America and, to a lesser extent, eastern Australia, for which there is growing evidence of Pleistocene extinctions (Jordan, 1997; Jordan et al., 2007; Sniderman et al., 2007). Fossil evidence suggests that extinction might explain the present‐day absence from one or more southern temperate regions of many vascular plant lineages. The southern temperate non‐vascular flora might have responded similarly to the environmental drivers of extinction of the vascular flora.

In this study, we infer the phylogeny of Lepidoziaceae using a multilocus data set that combines publicly available and newly generated molecular data. We perform a molecular dating analysis to infer the evolutionary timescale of the family, then use the dated tree to reconstruct its biogeographic history. Our study reveals the position of Micropterygioideae in the phylogeny of the Lepidoziaceae and provides an estimate of the divergence times and biogeographic history of the family.

## 2 MATERIALS AND METHODS

### 2.1 Molecular data set

To expand the taxonomic sampling of species of Lepidoziaceae, we sampled six herbarium specimens representing different species of *Micropterygium* from the Australian National Herbarium (CANB) (Appendix 1). About 25 mg of dried tissue was cleaned from each sample and, from these tissues, DNA was extracted by the Australian Genome Research Facility (Brisbane). Following PCR amplification and amplicon purification, dual‐direction Sanger sequencing of five markers by DNA BDT labelling reaction and capillary separation was carried out on an Applied Biosystems 3730xl Genetic Analyser (Appendix 2). We excluded sequences that did not show a close affinity with available sequences from Lepidoziaceae, as assessed using BLASTn searches. We combined the resulting 14 sequences from *Micropterygium* with sequence data from 141 species of Lepidoziaceae and 30 outgroup taxa available on GenBank, to produce a data set comprising a total of 177 taxa. We included the outgroup taxa *Herbertus* and *Lepicolea*, which have been shown to be close relatives of Lepidoziaceae (Cooper et al., 2012b; Feldberg et al., 2014). We also included outgroup taxa representing other members of Jungermanniales (*Plagiochila*, *Calypogeia* and *Scapania*) and Porellales (*Frullania*, *Acrolejeunea*, *Drepanolejeunea*, *Gackstroemia*, *Porella* and *Radula*) to provide nodes for fossil calibrations.

Our assembled data supermatrix included nucleotide sequences from seven chloroplast markers, four mitochondrial markers, and two nuclear markers (Appendix 3). This supermatrix had an occupancy of 44%, with 1013 sequences being available out of a possible 2301 (if sequences of all 13 markers had been available for all 177 taxa). We aligned the sequences of each of the 13 markers individually using MUSCLE (Edgar, 2004) and removed poorly aligned regions using Gblocks with less stringent selection (Castresana, 2000). We then tested for substitutional saturation and model adequacy using PhyloMAd (Duchêne et al., 2018, 2022). Based on entropy scores calculated using only the variable sites, we removed three sequence alignments that were found to carry a high risk of misleading phylogenetic inference (first codon sites of *psbA*, first codon sites of *rbcL* and *trnK*–*psbA* intergenic spacer).

### 2.2 Phylogenetic analyses and molecular dating

We performed a phylogenetic analysis using maximum likelihood in IQ‐TREE 2 (Bui et al., 2020), with the best‐fitting partitioning scheme selected using a greedy search (Appendix 4). The data set was partitioned into ten subsets, with each data subset allowed to evolve at a different relative rate, such that the branch lengths were proportionate across subsets (Duchêne et al., 2020). Node support values were estimated using 1000 bootstrap replicates.

Using Bayesian phylogenetic analysis, we jointly estimated the phylogeny and divergence times in BEAST v2.7.3 (Bouckaert et al., 2019) using a birth‐death tree prior and an uncorrelated lognormal relaxed clock (Drummond et al., 2006). Each data subset was allowed to have its own relative substitution rate. To calibrate the molecular clock, we specified a secondary calibration based on a previous age estimate for the split between Porellales and Jungermanniales (Laenen et al., 2014). We used a normal calibration prior with a mean of 319 Myr and a standard deviation of 32.65 Myr. In addition, we constrained the age of crown *Bazzania* to 34–381 Myr based on *Bazzania polyodus* (Feldberg et al., 2021), the only available fossil representing the family, and utilised 10 other fossil calibrations in the outgroup (Appendix 5). All of these fossils can be unambiguously assigned to extant genera, and many have previously been used for setting a minimum age constraint on crown groups of the genera and subgenera of the two orders (Cooper et al., 2012b; Feldberg et al., 2014; Heinrichs et al., 2007). The maximum age constraint chosen for all fossil calibrations is based on a previous date estimate for the Porellales–Jungermanniales split (Laenen et al., 2014), which had a 95% credible interval with an upper bound of 381 Ma.

We partitioned the alignments according to the scheme selected in IQ‐TREE and used Bayesian model averaging for all data subsets (Bouckaert & Drummond, 2017). The posterior distribution was estimated using Markov chain Monte Carlo sampling, with samples logged every 10^4^ steps over a total of 10^8^ steps. We ran the analysis three times and checked for sufficient sampling and convergence among the three chains using Tracer 1.7.1 (Rambaut et al., 2018). To examine any potential interactions among the calibration priors (Ho & Phillips, 2009), we ran an additional analysis in which we sampled from the prior distribution.

### 2.3 Biogeographical analyses

**FIGURE 2:**
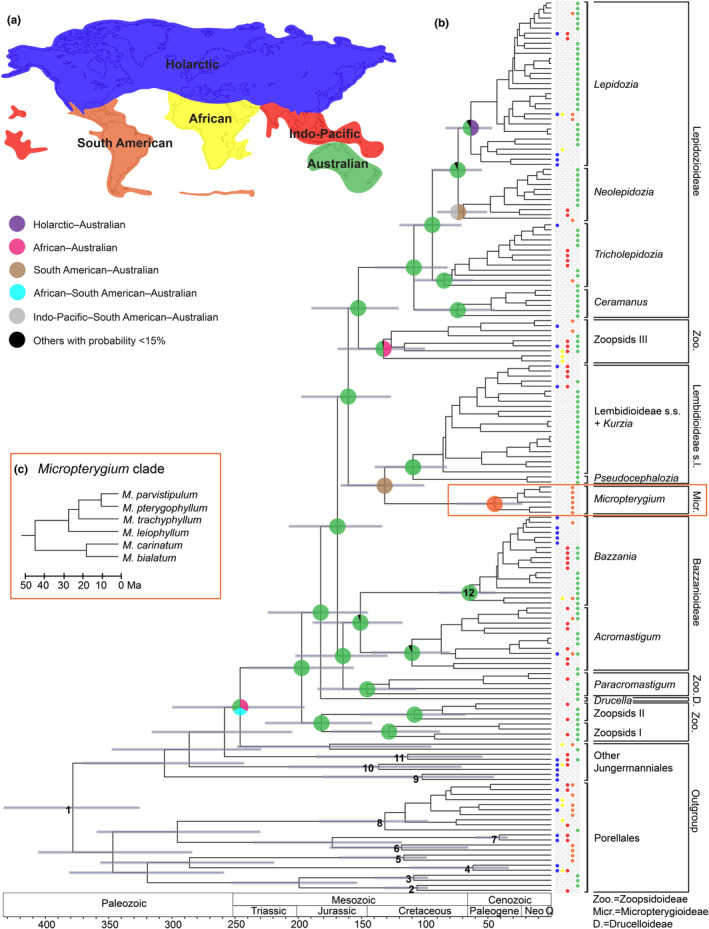
(a) Five floristic regions defined by Cox (2001). (b) Dated phylogenetic tree of Lepidoziaceae inferred using a Bayesian relaxed‐clock analysis of a multilocus data set. Circles at selected internal nodes show reconstructed ancestral ranges. Circles at the tips of the tree indicate present‐day distributions. Grey bars represent 95% credible intervals for the estimates of node ages. Numbers at internal nodes indicate the placement of calibrations for molecular dating: 1, secondary calibration based on Laenen et al. (2014); 2, *Radula* subg. *Amentuloradula* fossil; 3, *Radula* subg. *Odontoradula* fossil; 4, *Porella* fossil; 5, *Gackstroemia* fossil; 6, *Drepanolejeunea* fossil; 7, *Acrolejeunea* fossil; 8, *Frullania* fossil; 9, *Scapania* fossil; 10, *Calypogeia* fossil; 11, *Plagiochila* fossil; 12, *Bazzania* fossil. (c) Detailed view of the *Micropterygium* clade.

To investigate the historical biogeography of Lepidoziaceae, we obtained the distribution data of the species in the data set from authoritative literature sources, including taxonomic revisions and national flora treatments (Appendix 6). We assigned the taxa to five floristic regions based on Cox (2001): Holarctic, African, Indo‐Pacific, South American and Australian (Figure 2a). These floristic regions are based on different plant groups and take plate tectonics into account. The scheme is similar to the bryofloristic kingdoms by Schofield (1992), which also treated Australia and New Zealand as one region but merged the Indo‐Pacific region with a large portion of Africa. The ancestral locations were inferred using Statistical Dispersal‐Vicariance Analysis (S‐DIVA) (Yu et al., 2010) in the software package RASP (Yu et al., 2020), allowing a maximum number of five areas at each node, because the long evolutionary history of the family spans the movement of the continents. To account for phylogenetic uncertainty, we performed the ancestral state reconstruction on 10,000 trees sampled from the posterior distribution in addition to the maximum‐clade‐credibility tree from our Bayesian analysis.

## 3 RESULTS

### 3.1 Phylogeny and divergence times

**FIGURE 3:**
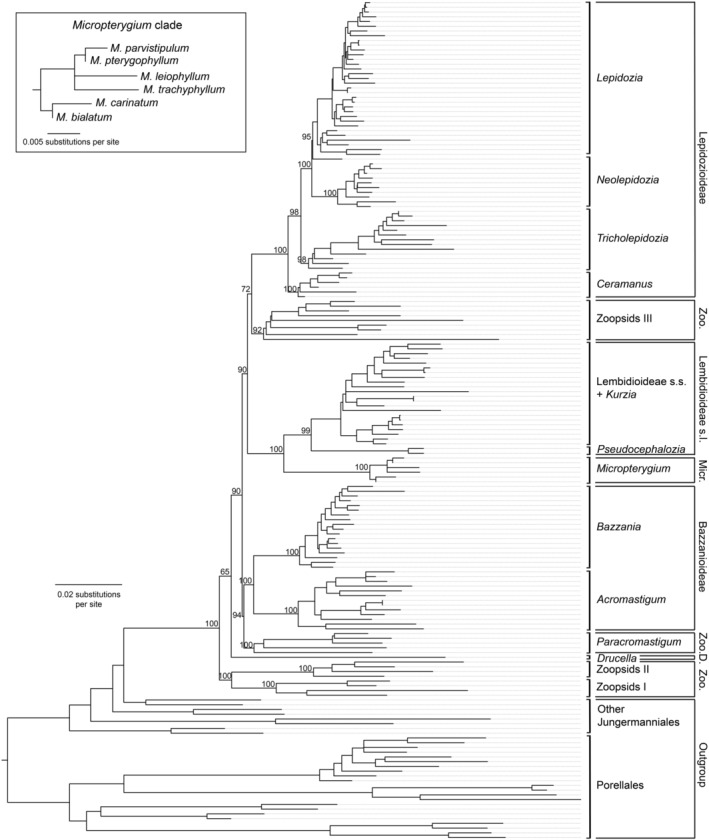
Maximum‐likelihood tree of Lepidoziaceae inferred using a multilocus data set. The major branches are labelled with bootstrap support values. The inset shows a detailed view of the *Micropterygium* clade (D., Drucelloideae; Micr., Micropterygioideae; Zoo., Zoopsidoideae).

Our phylogenetic analyses yielded well‐resolved trees for Lepidoziaceae with high bootstrap support and posterior probabilities for most nodes (Figures 2 and 3). Maximum‐likelihood and Bayesian analyses supported the monophyly of the family. The six species of *Micropterygium* form a sister clade to Lembidioideae. Overall, the maximum‐likelihood and Bayesian trees are similar to that inferred by Cooper et al. (2011) and support the same major clades, including Zoopsids I, Zoopsids II and Zoopsids III of the polyphyletic subfamily Zoopsidoideae.

Our results support the currently accepted circumscription of Lepidoziaceae (Schuster, 2000) as well as the revised circumscription presented by Cooper et al. (2012a) and Cooper (2013), where *Kurzia*, *Psiloclada* and some species of *Telaranea* are excluded from Lepidozioideae. Moreover, Zoopsidoideae do not form a monophyletic group, with its genera appearing in separate clades (Zoopsids I, Zoopsids II, Zoopsids III, *Neogrollea* and *Paracromastigum*). Bazzanioideae formed a monophyletic group with two distinct clades, *Acromastigum* and *Bazzania*. As in previous studies, some genera (including *Zoopsis*, *Telaranea*, and *Lembidium*) were found to be paraphyletic or polyphyletic.

The molecular dating analysis inferred a crown age for Lepidoziaceae of 197.3 Ma, with a 95% credible interval (CI) of 156.7–239.7 Ma (Figure 2b). The split between Zoopsids I and Zoopsids II occurred at 181.7 Ma (95% CI 142.2–225.7 Ma), whereas the divergence between Bazzanioideae and the *Paracromastigum* clade occurred at 164.6 Ma (95% CI 130.0–201.7 Ma). The divergence between *Acromastigum* and *Bazzania* occurred at 150.9 Ma (95% CI 118.2–188.0 Ma). More recently, *Lepidozia* and *Neolepidozia* diverged from each other at 73.6 Ma (95% CI 55.2–94.0 Ma), and the split between *Tricholepidozia* and the clade containing *Lepidozia* and *Neolepidozia* occurred at 93.2 Ma (95% CI 71.6–119.2 Ma).

### 3.2 Biogeographic reconstruction

Our reconstruction of ancestral location states placed the crown node of Lepidoziaceae in the Australian region and the divergence of the family from its sister lineage in three possible locations (Australian, African–Australian, and African–South American–Australian) (Figure 2b). Most of the major clades also originated in the Australian region. Crown Micropterygioideae emerged in South America. The analysis yielded more than one possible location for the origins of crown *Lepidozia* (Australian and Holarctic–Australian), crown *Neolepidozia* (South American–Australian and Indo‐Pacific–South American–Australian), and crown Zoopsids III (African–Australian and Australian).

## 4 DISCUSSION

### 4.1 Phylogeny and age of Lepidoziaceae

Our phylogenetic analysis of a multilocus data set has resolved the evolutionary relationships among major lineages within the family Lepidoziaceae, including the placement of the subfamily Micropterygioideae. The maximum‐likelihood and Bayesian trees inferred in our study are congruent with that of the most recent molecular phylogenetic study that used a large data set (Cooper et al., 2011), where the monophyly of the family was strongly supported. The same separate clades of Zoopsidoideae were inferred here, confirming the polyphyly of that subfamily.

Our molecular dating analysis, based on 11 fossil calibrations and one secondary calibration, yielded an age estimate for crown Lepidoziaceae of 197.3 Ma (95% CI 156.7–239.7 Ma). Our secondary calibration was based on a date estimate by Laenen et al. (2014), which we chose over other possible sources for secondary calibration because it represents the most comprehensive integration of fossil data (35 moss, 25 liverwort, and three hornwort fossils) and molecular data (eight markers: five chloroplast, two mitochondrial, and one nuclear) among all previous studies that estimated the divergence times among major liverwort groups. The posterior date estimate of the Porellales–Jungermanniales split, at 378.4 Ma (95% CI 326.2–432.5 Ma), is much older than that specified in the calibration prior (mean = 319; standard deviation = 32.65). This shift appears to be driven by a signal in the molecular data and the inclusion of a range of fossil‐based minimum age constraints across the tree but is not the product of interactions among the calibration priors (Appendix 7).

Our inferred divergence times for the family Lepidoziaceae are also much older than previous estimates. The analysis by Feldberg et al. (2014), which estimated the crown age of Lepidoziaceae at 174 Ma, used 20 fossil calibrations and a data set of 303 liverwort species (22 Lepidoziaceae). The phylogenetic tree was inferred using an unpartitioned analysis of *rbcL*. The study by Cooper et al. (2012b), which estimated the crown age of Lepidoziaceae at 116 Ma, used nine fossil calibrations and a data set comprising only three molecular markers from 212 liverwort species (64 Lepidoziaceae). Although that study used a partitioned data set, the absolute ages of the fossils were not used in the calibrations.

### 4.2 Evolutionary timescale and biogeographic history

The results of our molecular dating and biogeographic analyses allow us to propose an account of the evolutionary history of Lepidoziaceae. The crown node of the family has been placed after the break‐up of Pangaea but before the early fragmentation of Gondwana. Furthermore, many of the major lineages, including Bazzanioideae, Lepidozioideae, Lembidioideae, Zoopsids I, Zoopsids II, Zoopsids III and *Paracromastigum*, have estimated crown origins before Africa split from Antarctica during the mid‐Cretaceous (McLoughlin, 2001), leaving South America, Australia and New Zealand still connected to Antarctica (Smellie et al., 2020; van den Ende et al., 2017). These estimated ages suggest that these lineages of the family had sufficient time to spread throughout Gondwana before it began to break up. The results of our biogeographical analyses support an origin in land masses that were part of Gondwana, as postulated by Schuster (2000), considering that the inferred ancestral range of crown Lepidoziaceae is Australian and that the inferred possible locations of the divergence of the family from its sister lineage all show continents previously part of Gondwana (Figure 2b). Furthermore, many lineages (Zoopsids I, Zoopsids II, *Paracromastigum*, Bazzanioideae, Lembidioideae/*Kurzia* and Lepidozioideae) have inferred crown origins in the Australian region.

Although vicariance events could potentially account for the key divergences in Lepidoziaceae, the same cannot be said for some of the species‐level divergences. For instance, the lineage leading to *Z. argentea*, which occurs on Sunda in addition to Australia (part of Sahul) and New Zealand, diverged from *Z*. *nitida* about 92.1 Ma (52.8–134.1 Ma), much earlier than the formation of Sunda islands in the Miocene (Hall, 2002), and must have reached Sunda from Sahul by means of dispersal. The conditions in the mid‐Miocene until the present might have favoured floristic exchange between the two shelves through dispersal (Crayn et al., 2015). Although the estimated divergence of *Lepidozia* from *Neolepidozia* about 73.6 Ma (55.2–94.0 Ma) predates the separation between Australia and New Zealand (Veevers & McElhinny, 1976), the occurrence of *Lepidozia* in all five floristic regions is incompatible with vicariance. As supported by analysis of wind connectivity data, direction‐dependent long‐distance dispersal by wind rather than geographic proximity has been found to be responsible for floristic similarities in the Southern Hemisphere (Muñoz et al., 2004) where nearly all genera of Lepidoziaceae are present. Biological dispersers such as birds (Chmielewski & Eppley, 2019; Fife & de Lange, 2009; Lewis et al., 2014; Proctor, 1961) and bats, through faecal material (Parsons et al., 2007), might also have had a strong influence on past and present distribution patterns. Evidence of bryophyte diaspore in the plumage of transequatorial migratory birds is potentially connected to bipolar range expansions of some lineages (Lewis et al., 2014).

### 4.3 Micropterygioideae and its circum‐Antarctic links to Lepidoziaceae

Our analysis resolved the Neotropical endemic subfamily Micropterygioideae as the sister group to Lembidioideae and supports their status as a separate subfamily. A close relationship between Micropterygioideae and Lembidioideae is supported by morphological similarities mentioned by Schuster (2000). *Lembidium nutans* has loosely folded leaves resembling half canoes, similar to those of *Micropterygium*. The oil bodies in the species of both subfamilies are either reduced or completely lacking. *Micropterygium* was divided into two subgenera (Schuster, 2000), namely, subg. *Pseudolembidium* and subg. *Micropterygium*, without division into sections. All six species of *Micropterygium* in this study are in the latter subgenus, which is characterised by anisophyllous leaves. In another subgeneric classification scheme, the genus was divided into three sections (Reimers, 1933), namely, sect. *Conchifolia*, sect. *Subaequifolia*, and sect. *Genuina*. From the species included in the data set, *M. leiophyllum*, *M. parvistipulum*, *M. pterygophyllum* and *M. trachyphyllum* are all included in sect. *Genuina*, whereas the other two sections are unrepresented. *Micropterygium bialatum* and *M. carinatum*, which form a sister group to the rest of the genus, are not included in any of these sections. A more comprehensive sampling of the genus is needed to test the subgeneric classification schemes that have been proposed. The phylogenetic position of the monotypic genus *Mytilopsis* remains to be resolved. Further sampling of Lepidoziaceae will allow resolution of the remaining phylogenetic uncertainties.

The restricted range of Micropterygioideae in the Neotropical region stands in contrast with the wide distribution of the family as a whole, and this could, perhaps, be explained by the factors that limit dispersal, establishment of spores and population growth. Liverworts show no correlation between spore size and range (Laenen et al., 2016), but a strong correlation has been found between range and asexual reproduction. The lack of asexual reproduction in the subfamily (Schuster, 2000), as well as its relatively young crown age, also provide potential explanations for its limited geographic distribution. It is also possible that the lineage once occupied the Australian region and then went extinct there at some point. The results of our phylogenetic dating analysis support this possibility. We estimated that Micropterygioideae split from Lembidioideae 131.6 Ma (101.1–165.8) in the Australian and South American regions of Gondwana during a time when Antarctica was still connected to Africa, South America, and Australia. The estimated crown origin of the subfamily is 44.6 Ma (95% CI 23.3–73.9 Ma) in South America, suggesting that it occurred before South America separated from Antarctica 30 Ma (van den Ende et al., 2017). These circum‐Antarctic links of the subfamily to the rest of the family, through the Micropterygioideae–Lembidioideae split during the early Cretaceous, strongly suggest extinction of the lineage in the region, but this requires verification through fossil evidence. However, bryophyte fossils are rarely found due to the low likelihood of preservation in the form of cuticles, compressions, charcoals, amber inclusions, or permineralizations (Tomescu et al., 2018).

Neotropical endemic taxa either have origins in the Neotropics itself, e.g., Calyceraceae (Brignone et al., 2023), or from elsewhere, e.g., Cyclanthaceae (Leal et al., 2022) and Cannaceae (Kress & Specht, 2006). In Cyclanthaceae, a fossil of a representative (*Cyclanthus*) has been found in Europe which is outside the present‐day range of the family. In Cannaceae, no fossil has been found outside the present‐day range, but analyses of the biogeographic origin and diversification of Zingiberales revealed that the family diversified in Africa or tropical America. Our study shows that Micropterygioideae are possibly among the lineages that the Neotropical region holds in its collection of taxa with circum‐Antarctic links. There are several other Lepidoziaceae taxa that are exclusive to the Neotropics, including *Protocephalozia* of the monotypic Protocephalozioideae and some elements of the heterogeneous Zoopsidoideae (*Monodactylopsis*, *Odontoseries*, and *Pteropsiella*). Including these taxa in the phylogenetic dating of Lepidoziaceae will reveal their biogeographical connection to the rest of the family. Since these genera are also Neotropical endemics, resolving their positions in the phylogeny of the family and their estimated ages will also give hints about how they reached the Neotropics.

### 4.4 Conclusions

Our study has presented a reconstruction of the evolutionary and biogeographic history of the liverwort family Lepidoziaceae, including the phylogenetic placement of the subfamily Micropterygioideae. Key divergences can be explained by vicariance, but long‐distance dispersal is likely to have played a large role in the recent diversification of the family. However, the phylogeny of Lepidoziaceae is still not fully resolved. Improved resolution of phylogenetic relationships and biogeographic history can potentially be achieved through more comprehensive taxon sampling, with genetic data yet to be obtained from several genera in the family. To allow confident taxonomic revision, it will be necessary to conduct further phylogenetic analysis using larger numbers of markers, such as those obtained by exon capture, transcriptomics or even whole‐genome sequencing.

## AUTHOR CONTRIBUTIONS

**Antonio L. Rayos Jr.:** Conceptualization (equal); data curation (equal); formal analysis (equal); funding acquisition (equal); investigation (equal); methodology (equal); project administration (equal); validation (equal); visualization (equal); writing – original draft (lead); writing – review and editing (equal). **Matthew A. M. Renner:** Conceptualization (equal); methodology (equal); supervision (equal); validation (equal); visualization (equal); writing – review and editing (equal). **Simon Y. W. Ho:** Conceptualization (equal); formal analysis (equal); funding acquisition (equal); methodology (equal); supervision (equal); validation (equal); visualization (equal); writing – review and editing (equal).

## Data Availability

The DNA sequences that support the findings of this study are available in GenBank of NCBI at https://www.ncbi.nlm.nih.gov/ under the accession numbers provided in Appendix 3.

## APPENDIX 1

*Micropterygium* samples from the Australian National Herbarium (CANB) used in this study.

**Table:**

Species	Collection number	Accession number
*M. bialatum*	Boom and Gopaul 7447	CANB 00906825
*M. carinatum*	Halling 5534	CBG 9210343
*M. leiophyllum*	Prance et al. 15566	CBG 8205487
*M. parvistipulum*	Prance et al. 11404	CBG 8205491
*M. pterygophyllum*	Buck 11370	CANB 00785805
*M. trachyphyllum*	Boom and Gopaul 7240	CANB 00906826

## APPENDIX 2

Molecular markers amplified and sequenced for the *Micropterygium* samples in this study.

**Table:**

Marker	Genome	Primer sequences (5′–3′)	Reference
*trnL*‐*trnF*	Chloroplast	F: ATTTGAACTGGTGACACGAG R: CGAAATCGGTAGACGCTACG	Taberlet et al. (1991)^b^
*trnG* intron	Chloroplast	F: ACCCGCATCGTTAGCTTG or ATTCGGTGATTTAGTTACG^a^ R: GCGGGTATAGTTTAGTGG	Pacak and Szweykowska‐Kuliñska (2000)^c^
	Chloroplast		
*psbA*‐*trnH*	Chloroplast	F: GTTATGCATGAACGTAATGCTC R: CGCGCATGGTGGATTCACAATCC	Stech et al. (2011)^d^
*nad1*	Mitochondrial	F: GCATTACGATCTGCAGCTCA R: GGAGCTCGATTAGTTTCTGC	Sun (2002)^e^
26S rDNA	Nuclear	F: GAGTCGGGTTGTTTGGGA R: TTGGTCCGTGTTTCAAGACG	Kuzoff et al. (1998)^f^

^a^ Forward primer redesigned to target shorter amplicon for a higher chance of success.

^b^ Taberlet, P., Gielly, L., Pautou, G., & Bouvet, J. (1991). Universal primers for amplification of three non‐coding regions of chloroplast DNA. *Plant Molecular Biology*, 17, 1105–1109.

^c^ Pacak, A., & Szweykowska‐Kuliñska, Z. (2000). Molecular data concerning alloploid character and the origin of chloroplast and mitochondrial genomes in the liverwort species *Pellia borealis*. *Journal Of Plant Biotechnology*, 2, 101–108.

^d^ Stech, M., Kolvoort, E., Loonen, M. J. J. E., Vrieling, K., & Kruijer, J. D. (2011). Bryophyte DNA sequences from faeces of an arctic herbivore, barnacle goose (*Branta leucopsis*). *Molecular Ecology Resources*, 11, 404–408.

^e^ Sun, G. L. (2002). Interspecific polymorphism at non‐coding regions of chloroplast, mitochondrial DNA and rRNA IGS region in *Elymus* species. *Hereditas*, 137, 119–124.

^f^ Kuzoff, R. K., Sweere, J. A., Soltis, D. E., Soltis, P. S., & Zimmer, E. A. (1998). The phylogenetic potential of entire 26S rDNA sequences in plants. *Molecular Biology and Evolution*, 15, 251–263.

## APPENDIX 3

Data supermatrix (MicrosoftExcelfileprovided).

## APPENDIX 4

Partitioning scheme used for maximum‐likelihood and Bayesian phylogenetic analyses.

**Table:**

Subset	Substitution model	Markers
1	TN + F + I + G4	26S rRNA, *atpB* codon position 1
2	GTR + F + I + G4	5.8S rRNA, *nad1*
3	GTR + F + G4	ITS1, ITS2
4	GTR + F + I + G4	*atpB* codon position 2, *psbA* codon position 2, *rbcL* codon position 2
5	TVM + F + I + G4	*atpB* codon position 3, *rbcL* codon position 3, *rps4* codon position 3
6	GTR + F + I + G4	*nad5*‐*nad4*, *nad5*, *rps4* codon position 1, *rps4* codon position 2
7	TIM + F + I + G4	*psbA*‐*trnH* intergenic spacer, *trnG* intron
8	GTR + F + I + G4	*psbA* codon position 3
9	K3Pu + F + I + G4	*psbT*‐*psbH*, *trnL*‐*trnF*
10	GTR + F + I + G4	*rps3*

## APPENDIX 5

Fossils used for age calibrations based on Feldberg et al. (2021).

**Table:**

Fossil	Age assignment	Corresponding taxon	Origin and age
*Drepanolejeunea eogeana*	15–381	*Drepanolejeunea* crown	La Toca Formation, Dominican Republic; 15–20 Ma
*Calypogeia stenzeliana*	34–381	*Calypogeia* crown	Baltic region; 34–41 Ma
*Porella subgrandiloba*	34–381	*Porella* crown	Baltic region; 34–41 Ma
*Bazzania polyodus*	34–381	*Bazzania* crown	Baltic region; 34–41 Ma
*Plagiochila groehnii*	34–381	*Plagiochila* crown	Baltic region; 34–41 Ma
*Scapania hoffeinsiana*	34–381	*Scapania* crown	Baltic region; 34–41 Ma
*Acrolejeunea ucrainica*	35–381	*Acrolejeunea* crown	Klesov, Ukraine; 35–37 Ma
*Gackstroemia cretacea*	99–381	*Gackstroemia* crown	Northern Myanmar; 99 Ma
*Frullania baerlocheri*, *F. cretacea*, *and F. partita*	99–381	*Frullania* crown	Northern Myanmar; 99 Ma
*Radula cretacea*	99–381	*Radula* subg. *Odontoradula* crown	Northern Myanmar; 99 Ma
*Radula heinrichsii*	99–381	*Radula* subg. *Amentuloradula* crown	Northern Myanmar; 99 Ma

## APPENDIX 6

Distribution data (MicrosoftExcelfileprovided).

## APPENDIX 7

Comparisons of marginal prior densities (blue) and marginal posterior densities (red) for the ages of four nodes in the phylogenetic tree. (a) *Drepanolejeunea* crown, (b) *Bazzania* crown, (c) *Radula* subg. *Odontoradula* crown and (d) Porellales–Jungermanniales split.
