# Correction to “The Neotropical endemic liverwort subfamily Micropterygioideae had circum‐Antarctic links to the rest of the Lepidoziaceae during the early Cretaceous”

**DOI:** 10.1002/ece3.11199

**Published:** 2024-04-08

Rayos, A.L., Jr., M.A.M. Renner, and S.Y.W. Ho. 2024. The Neotropical endemic liverwort subfamily Micropterygioideae had circum‐Antarctic links to the rest of the Lepidoziaceae during the early Cretaceous. Ecology and Evolution 14: e11066.

The link attached to Appendix 3 directs to the Excel file that is supposed to be for Appendix 6. The correct link for Appendix 3 is: https://docs.google.com/spreadsheets/d/1MWUVFd9Dk3wNh90I5XJPiEeQtORnlZwXCKOsh2qU1Ow/edit#gid=0.

We apologize for this error.

